# Effects of intratumoral microbiota on tumorigenesis, anti-tumor immunity, and microbe-based cancer therapy

**DOI:** 10.3389/fonc.2024.1429722

**Published:** 2024-09-26

**Authors:** Jingwei Zheng, Hao Chen

**Affiliations:** Department of Pathology, Hospital for Skin Diseases, Institute of Dermatology, Chinese Academy of Medical Sciences and Peking Union Medical College, Nanjing, China

**Keywords:** intratumoral microbiota (IM), tumor microenvironment (TME), fecal microbiome transplantation (FMT), engineered bacteria, bacteriophage, oncolytic virus

## Abstract

Intratumoral microbiota (IM) has emerged as a significant component of the previously thought sterile tumor microenvironment (TME), exerting diverse functions in tumorigenesis and immune modulation. This review outlines the historical background, classification, and diversity of IM, elucidating its pivotal roles in oncogenicity, cancer development, and progression, alongside its influence on anti-tumor immunity. The signaling pathways through which IM impacts tumorigenesis and immunity, including reactive oxygen species (ROS), β-catenin, stimulator of interferon genes (STING), and other pathways [NF-κB, Toll-like receptor (TLR), complement, RhoA/ROCK, PKR-like ER kinase (PERK)], are discussed comprehensively. Furthermore, we briefly introduce the clinical implications of IM, emphasizing its potential as a target for novel cancer therapies, diagnostic biomarkers, and prognostic indicators. Notably, microbe-based therapeutic strategies such as fecal microbiome transplantation (FMT), probiotics regulation, bacteriotherapy, bacteriophage therapy, and oncolytic virotherapy are highlighted. These strategies hold promise for enhancing the efficacy of current cancer treatments and warrant further exploration in clinical settings.

## 1 Introduction

The tumor microenvironment (TME), encompassing cancer cells, stromal cells, immune cells, and soluble factors such as cytokines and chemokines, plays a pivotal role in cancer progression and therapeutic outcomes. Understanding the regulatory mechanisms governing the immune compartment within the TME is crucial for advancing cancer treatment strategies. Recently, the discovery of intratumoral microbiota (IM) has added a new dimension to our understanding of the TME (1–3). Despite its low biomass ranging from 0.1% to 10%, IMs have emerged as significant contributors to the tumor ecosystem, profoundly influencing tumorigenesis, cancer progression, and responses to cancer therapy (4).

Over the past decades, propelled by technological advancements, researches into IMs have revealed common characteristics and highlighted their complex interplay within the TME. The implications of IM extend beyond mere association; they have been implicated in tumor diagnosis, prognosis, and treatment guidance (1–3). As the field rapidly evolves, an updated review is warranted to consolidate current knowledge and identify emerging trends.

n this review, we first provide a concise overview of the historical context, classification, and diversity of IM. We then introduce their roles in carcinogenesis, cancer development, and progression, emphasizing their influence on anti-tumor immunity and the underlying signaling pathways. Furthermore, we discuss the clinical implications of IM, including their potential as targets for cancer therapy, diagnostic biomarkers, and prognostic indicators. Finally, we propose innovative microbe-based strategies for cancer treatment aimed at broad clinical applications. By synthesizing these insights, this review aims to underscore the transformation potential of IM research in reshaping our approach to cancer management.

## 2 The history, classification, and diversity of intratumoral microbiota

The concept of IMs as integral components of the TME has evolved significantly over time, tracing its roots back to discoveries made in the mid-19th century. Early microbiologists identified live microorganisms within tumors, yet due to technological limitations and prevailing skepticism, these findings initially faced resistance in the scientific community. A pivotal breakthrough occurred in 1911 with the discovery of the Rous Sarcoma virus, marking one of the first recognized examples of oncogenic viruses within tumors (5). Subsequent decades witnessed the identification of various viruses linked to cancer, such as Epstein-Barr virus (EBV), Hepatitis B virus (HBV), Hepatitis C virus (HCV), and Human papillomavirus (HPV). These discoveries underscored the diverse viral contributions to tumorigenesis. The identification of bacteria as oncobacteria gained prominence following the groundbreaking revelation in 1983 of *Helicobacter pylori (H. pylori)* as a causative agent in gastric cancer (3, 6). Beyond bacteria, *Chlamydia trachomatis (C. trachomatis)* emerged as another microbial species implicated in the pathogenesis of cervical cancer, squamous cell carcinoma, and ovarian cancer (7). Recent advancements, exemplified by Nejman et al.’s comprehensive analysis in 2020, have expanded the understanding of IMs to include a wide spectrum of bacterial species across various human tumor types (8). Concurrent studies by Narunsky-Haziza and Dohlman et al. further elucidated the role of fungi within tumors, highlighting their potential impacts on cancer diagnosis and prognosis (9, 10).

In addition to viruses and bacteria, the IM spectrum encompasses bacteriophages and protozoa, though these are not extensively discussed in this review. Notably, the composition and abundance of IMs exhibit significant variability within different cancer types, subtypes, and stages. For instance, lung cancer exhibits a distinct microbial colonization influenced by environmental factors, whereas breast cancer harbors a remarkably diverse microbiome (8, 11). Huang et al. highlighted higher levels of *Gammaproteobacteria* in cancerous tissues compared to normal tissues, along with significant increases in *Streptococcaceae* and *Lactococcus* in cirrhotic hepatocellular carcinoma (HCC), suggesting their potential as biomarkers (12). *Fusobacterium* and *enterotoxigenic Bacteroides fragilis* (ETBF) have implications in colorectal cancer (CRC) progression. CRC tissues exhibit higher abundances of *Lactococcus, Bacteroides, Fusobacterium, Prevotella*, and *Streptococcus*, while *Pseudomonas* and *E. coli-Shigella* are enriched in adjacent normal tissues (13). Moreover, studies have indicated varying intratumor microbiome profiles in cancer recurrence, emphasizing the dynamic nature of IMs in cancer progression. In conclusion, understanding the historical context, diverse classifications, and dynamic nature of intratumoral microbiota is crucial for unraveling their intricate roles in carcinogenesis and disease progression across different cancer types.

## 3 The roles of IMs in tumorigenesis, cancer development and progression

Bacteria associated with cancer have long been regarded as being opportunistic, but it became clear in the late 20th century that bacteria can directly cause cancer (5). An oncogenic bacterial “driver-passenger” model has been proposed to explain the role of IMs in tumorigenesis (14–16). Oncoviruses such as HPV, HBV, and HCV can also act as “drivers,” exerting various effects on cancer immunosurveillance and cancer progression. Herein, we focus on bacteria as examples to elucidate this model.

Endogenous “driver bacteria,” such as *Escherichia coli* group B2 strains capable of producing enterotoxin-induced single-stranded DNA breaks, can aggregate into non-anchor colonies within infected cells, thereby increasing mutation frequencies (17). Certain microbial metabolites, like hydrogen sulfide (H2S) produced by resident sulfate-reducing bacteria such as *Fusobacterium nucleatum* and *E. coli*, generate reactive oxygen species (ROS) that disrupt host DNA (18). Pathogens like *Chlamydia trachomatis* and *Helicobacter pylori*, associated with gynecological and gastric cancers respectively, inhibit homologous recombination and induce error-prone non-homologous end-joining, thereby compromising host DNA repair processes. Other bacteria such as *Hungatella hathewayi* and *Fusobacterium nucleatum* induce epigenetic modifications of the host genome by upregulating DNA methyltransferases through mechanisms that are not fully understood, leading to hypermethylation and silencing of tumor-suppressor genes (including *CDX2* and *MLH1*), thereby promoting tumorigenesis (19–21). *Enterotoxigenic Pseudomonas fragilis (ETPF)* can sustain persistent Th-17-type inflammatory stimulation, gradually decreasing in number due to this stimulation and being replaced by opportunistic “passenger bacteria” that can dominate the existing ecological niche, disrupt local innate immunity, and ultimately contribute to tumor progression (22). *Staphylococcus haemolyticus (S. haemolyticus)* is considered a “passenger” bacterium, promoting epithelial cell differentiation and forming biofilms on exposed basement membrane collagen fibers within disrupted colonic wall structures (23). Models explain the occurrence of *Clostridium*-induced CRC, a silent disease affecting various age groups worldwide, with heightened mucosal biofilm levels harboring specific microbial species interacting with mucosal immune and microbial communities, imparting partial oncogenic properties (24). 5-fluorouracil (5-FU), a first-line chemotherapeutic agent for CRC, can serve as an inhibitor of *F. nucleatum* isolates from CRC but does not work for CRC-isolated *E. coli* with the capacity to modify 5-FU. When *E. coli* exists, it attenuates 5-FU virulence against *F. nucleatum* and human CRC epithelial cells which are otherwise sensitive to 5-FU, thereby leading to resistance to chemotherapeutic agents and promoting tumor progression (25). Collectively, these studies underscore a robust link between intratumoral microbiota and carcinogenesis, as well as cancer progression.

**Figure 1:**
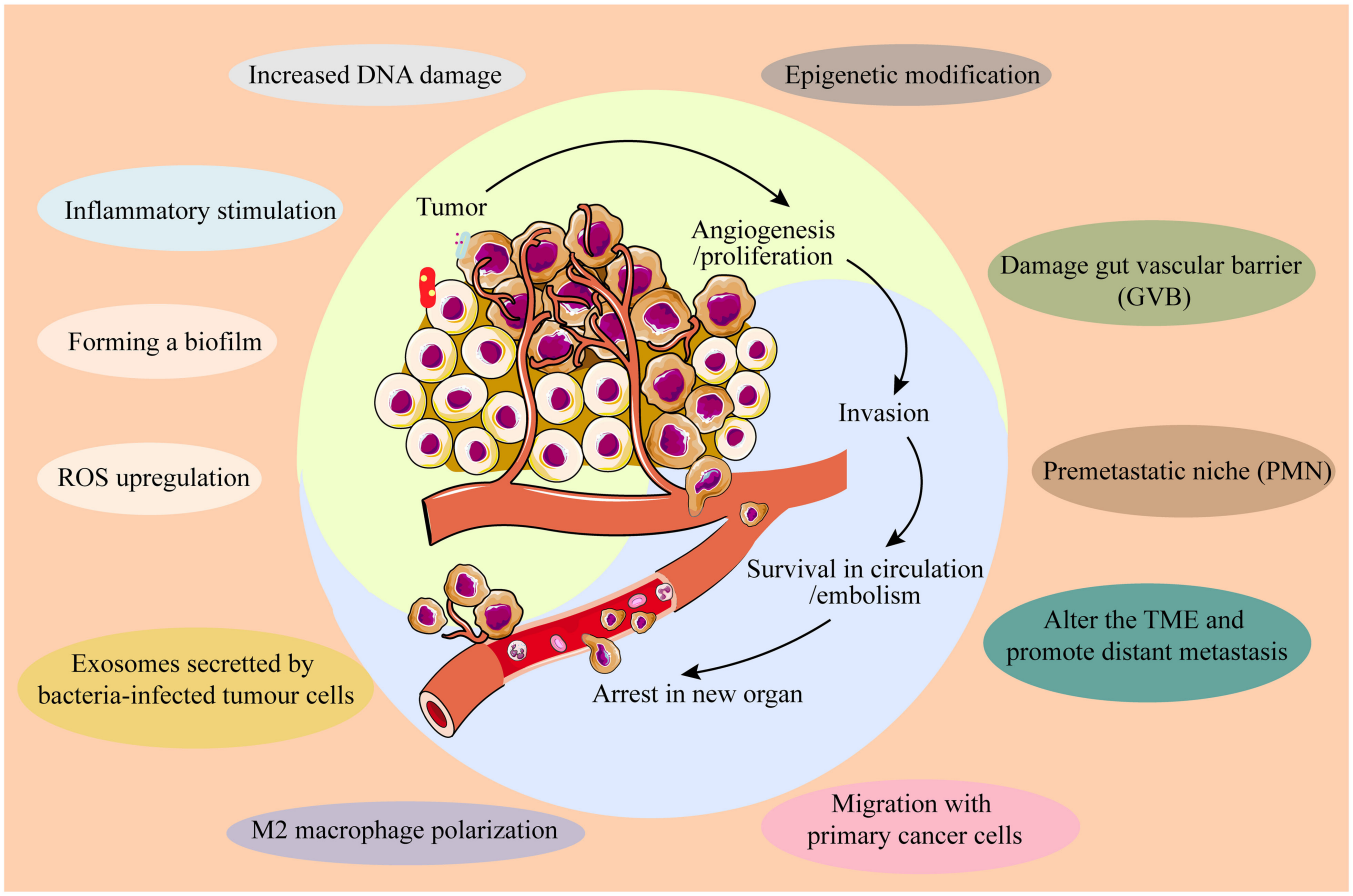
IM influences cancer initiation, development, progression, and metastasis through diverse mechanisms.

Tumor metastasis remains the leading cause of cancer-related mortality. IM contributes to cancer metastasis through multiple mechanisms across various cancer types: ① Co-migration with primary cancer cells: *Fusobacterium* has been detected in both primary tumors and matched distant metastases of colorectal cancer (26). ② Disruption of the gut vascular barrier (GVB): pathogens such as *Salmonella typhimurium* can disrupt the GVB, facilitating translocation and toxin transmission. For instance, VirF+ *E. coli* within tumors can degrade the GVB, allowing gut microbes to translocate to the liver and establish a “premetastatic niche” (PMN), enhancing chemotactic factor expression and extracellular matrix deposition, and upregulating plasmalemma vesicle-associated protein 1 (PV1) (27). ③ Modification of the TME to promote distant metastasis: *Fusobacterium nucleatum* infection induces colorectal cancer cells to release exosomes enriched with CXCL16/RhoA/IL-8 and miR-1246/92b-3p/27a-3p, which are transferred to uninfected CRC cells, thereby promoting liver metastasis (28). Additionally, *Fusobacterium nucleatum* induces M2 macrophage polarization within the TME, promoting CRC liver metastasis by upregulating chemokine CCL20 and ICAM1 expression (29). Most recurrences of lung cancer (LC) may be due to an abundance of butyrate-producing bacteria which promotes LC metastasis by inhibiting HDAC2 and increasing H3K27 acetylation at the H19 promoter and inducing M2 macrophage polarization (30). Altogether, IM profoundly influences cancer initiation, development, progression, and metastasis through diverse mechanisms (**Figure 1**), while also impacting anti-tumor immunity.

## 4 The impacts of IMs on anti-tumor immunity

Intratumoral microbiota has different effects on the tumor immune response, some with cancer-promoting effects and others with tumor suppressors (**Table 1**).

**Table 1:** Mechanisms of IMs in anti-tumor immunity.

	Mechanisms	Associated IMs
Promoting anti-tumor immunity	Promoting antigen presentation	① *Bifidobacteria* (3, 31) ② *Akkermansia muciniphila* (31) ③ HPV (32)
	Activation of immune effector cells	① *Lachnoclostridium, Gelidibacter and Flammeovirga* (33) ② EBV, HBV, and MCPyV (3) ③ *Saccharopolyspora, Pseudoxanthomonas and Streptomyces* (34) ④ *Clostridium* (35) ⑤ B*ifidobacterium* (24)
	Metabolites	① *Clostridium* (36) ② *Mycobacteria* (37)
	Formations of TA-TLSs	① *Hepatocephala* spp (3). ② VSV, NDV, or HSV-1 (38–40) ③ Adenovirus with mIL-15 (39)/mTNFα- and mIL-2 (38)
Inhibiting anti-tumor immunity	ROS upregulation	② *B. fragilis* (3) ④ *F. nucleatum* (41)
	Inactivation/dysregulation of immune effector cells	① *F. nucleatum* (42, 43) ② *Methylobacterium* (44)
	A proinflammatory environment	① Fusobacterium, Streptococcus, and Peptostreptococcus (41, 45) ② *Enterococcus and Escherichia/Shigella* (46) ③ *H. pylori* (47) ④ *Aspergillus sydowii* (11) ⑤ A*lternaria alternata* (48)
	A tumor immunosuppressive microenvironment	① HBV, HCV, *S. aureus, N. ramosa*, and *HPV* (49) ② *Lactobacillus* (50)
	Others	*Malassezia* spp (51).

IM, Intratumoral microbiota; EBV, Epstein-barr virus; HBV, Hepatitis B Virus; MCPyV, Merkel cell polyomavirus; TA-TLSs, Tumor-associated tertiary lymphoid structures; ROS, Reactive oxygen species;HCV, Hepatitis C Virus.

### 4.1 Strengthening anti-tumor immune response

**Figure 2:**
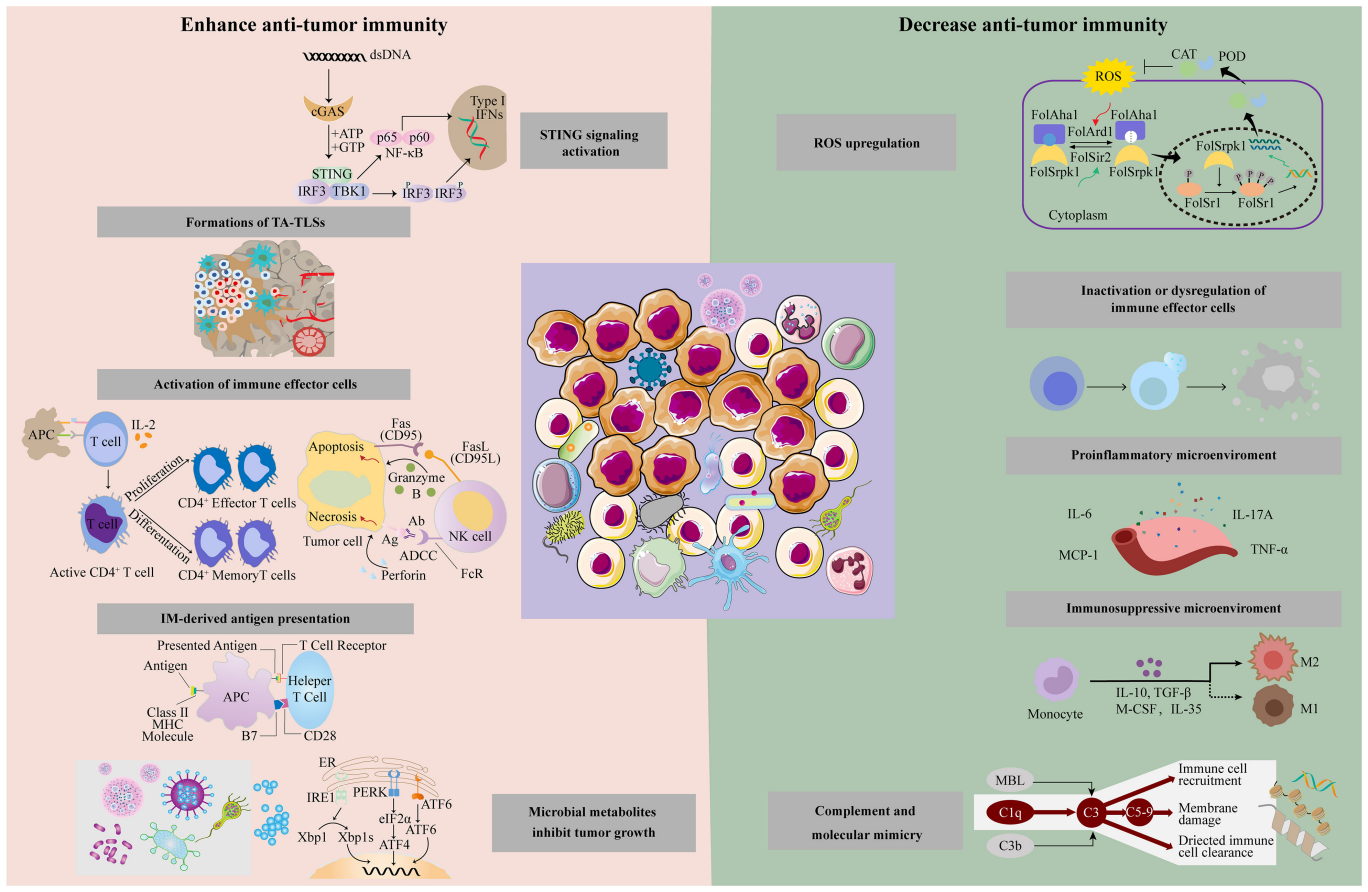
Intratumoral microbiota has different effects on the tumor immune response through different mechanisms, including enhanced anti-tumor immune response and suppression of anti-tumor immunity.

IMs boost anti-tumor immunity by augmenting stimulator of interferon genes (STING) signal and antigen presentation, activating immune effector cells and forming a tumor-suppressive microenvironment (**Figure 2**).

#### 4.1.1 Promoting STING signal and IM-derived antigen presentation

Microbe-associated molecular patterns may stimulate pattern recognition receptors (PRRs) and intensify immune responses based on cross-reactivity between tumors and microbial antigens. Microbiota can activate the STING signaling pathway, which improves the cross-priming of dendric cells (DCs) and further induces naive T cell proliferation and differentiation to promote anti-tumor responses (52). *Bifidobacteria* can migrate to and invade intestinal cancer cells. Following anti-CD47 targeted immunotherapy, topically and systematically administered *Bifidobacteria* initiate STING signaling to coordinately kill tumor cells (3). Analogously, STING activators derived from *Akkermansia muciniphila* induce the release of interferon-I (IFN-I) from intratumoral mononuclear cells, polarize macrophages, and promote the cross-talk with natural killer (NK) cells and DCs (3, 31).

In addition to triggering more potent host antigen presentation through the aforementioned mechanisms, the antigen-presenting cell (APC)-presented peptides of intratumoral bacteria provoke specific T cell responses targeting tumor cells for the microbes mostly located within tumor cells and immune cells. Intratumoral injection of bacteria or their antigens provides immunostimulatory effects to combat tumors, similar to Coley toxin, a heat-inactivated mixture of *Serattia marcescence* and *Streptococcus pyogenes*. HPV E2 and E5 are major targets of the intratumoral CD8^+^ T cell response in patients with HPV-positive head and neck squamous cell carcinoma (HNSCC). Thus, therapeutic vaccination strategies for HPV-positive patients should also consider including E2 and E5 as vaccine antigens to elicit tumor-reactive CD8^+^ T cell responses of maximal breadth (32).

#### 4.1.2 Activation of immune effector cells

Some IMs are positively correlated with activated and infiltrated immune effector cells and promote anti-tumor immunity through mechanisms such as activation of STING and IFN signaling pathway, regulation of cytokines or chemokines, initiation of endoplasmic reticulum (ER) stress, and other potential mechanisms.

In cutaneous malignant melanoma (MM), intratumoral *Lachnoclostridium*, *Gelidibacter*, and *Flammeovirga* were the top three genera positively associated with CD8^+^ T cell infiltration. They are also positively correlated with the secretion of chemokines such as CXCL9, CXCL10, and CCL5 (33). Oncoviruses such as EBV, HBV, and Merkel cell polyomavirus (MCPyV) can also induce chemokine production in tumor tissues, and then increase CD8^+^T cell infiltration to prolong patient survival (3). In addition, a higher α diversity in Ims and density of CD8 ^+^ T cells and granzyme ^+^ B cells in the TME are associated with long-term survival (LTS) in pancreaticductal adenocarcinoma (PDAC) patients, although the levels of macrophages, Tregs, and MDSC cells exhibited no significant difference from those in short-term survival patients, indicating *Saccharopolyspora, Pseudoxanthomonas*, and *Streptomyces* can recruit and activate CD8^+^ T cells in PDAC tissues (34). The exogenous administration of microbes has also been found to affect immune cell infiltration. For example, *bifidobacterium* incorporation into cancers facilitates NK cell activation (35), and four commensal *Clostridiales* strains (CC4) orally administered to mice can accumulate more CD8^+^ T cells (24).

#### 4.1.3 Microbial metabolites inhibit tumor growth

Trimethylamine-N-oxide (TMAO) released by *Clostridium* initiates PKR-like ER kinase (PERK)-mediated ER stress, induces pyroptosis, and augments CD8 ^+^ T cell-mediated tumor killing (36). A significantly increased fungal mass in HCC was negatively associated with carbohydrate antigen199 (CA199) levels. Metabolomic and transcriptomic analyses have deduced that mycobacteria interfere with tumor progression via amino acid metabolism (37).

#### 4.1.4 Formations of tumor-associated tertiary lymphoid structures

Tertiary lymphoid structures (TLSs) are ectopic lymphoid aggregates developing within non-lymphoid tissues, which exhibit phenotypic and functional similarities with conventional secondary lymphoid organs (SLOs). TLSs are not typically present under normal physiological conditions but have been noted in various pathological states, such as autoimmune diseases, chronic infections, and cancers. Tumor-associated TLSs (TA-TLSs) are associated with improved patient survival by promoting lymphocyte infiltration and activation in the TME to arouse anti-tumor immunity during maturation (53, 54). Some IMs or exogenous microorganisms contributed to TA-TLS formation. *Hepatocephala* spp. enhance TLS development around tumors and inhibit intestinal cancer (3). Natural oncolytic viruses (OVs) can also induce TLSs. For instance, after infecting tumor-bearing mice with vesicular stomatitis virus (VSV), Newcastle disease virus (NDV), or herpes simplex virus (HSV-1), antiviral memory T cells were reactivated after being immunized secondly, favoring mutual reinforcement of antiviral and anti-tumor activities to form TLSs, thus eliciting larger-range adaptive immunity compared to controls (38–40). In immune checkpoint inhibitor (ICI)-naïve mice with refractory oral carcinoma, the therapeutic efficacy of anti-PD-1/PD-L1 combined with the mTNFα- and mIL-2-carrying non-replicative adenovirus was improved along with the upregulated expression of a TLS relative gene signature (38). A mIL-15 armed-oncolytic adenovirus recruited activated T and NK cells through STING-TBK1-IRF3-mediated DC activation, prompting vascular normalization and TLS formation (39). These studies showed that microbe-induced TA-TLSs could be a new strategy for cancer immunotherapy.

### 4.2 Inhibiting antitumor immune responses

Some IMs can counteract effective immunotherapy via releasing ROS, inactivating immune effector cells, promoting inflammation, and forming immunosuppressive microenvironments (**Figure 2**).

#### 4.2.1 ROS upregulation

Symbiotic microorganisms can produce ROS, contributing to tumor progression in multiple ways (55), including the regulation of immune responses. The bacterium enterotoxigenic *Bacteroides fragilis* (ETBF) causes pre-inflammation or immunosuppression via ROS and DNA damage in the TME, resulting in colorectal cancer (3). In the gastrointestinal tract, activation of the Toll-like receptor (TLR4)-ROS and nitric oxide dioxygenase (NOD)-like receptor (NOD1/2)-dependent signaling by *F. nucleatum* enriches neutrophil extracellular trap (NET) formation, thus, tumors progress and metastasize through angiogenesis, Epithelial-Mesenchymal Transition (EMT), matrix metalloproteinase (MMP)-mediated basement membrane protein degradation, and the capture of CRC cells (41).

#### 4.2.2 Inactivation or dysregulation of immune effector cells

Some IMs have a negative correlation with tumor-infiltrating T cell density and can cause T cell dysfunction and favor tumor growth and metastasis. Transcriptome and digital pathological analyses revealed that the bacterial load within breast cancer (BC) hampered T cell infiltration, with *F. nucleatum* being negatively correlated, especially with CD3^+^T cells (42). *F. nucleatum* inoculation inhibited the aggregation of tumor-infiltrating T cells and recruited tumor-associated macrophages (TAMs) and fibroblasts (43). Conversely, antibiotic treatment counteracts *F. nucleatum*-induced side effects, thereby confirming its detrimental effects on immune effector cells (42). Intratumoral *Methylobacterium* induces dysfunction of the memory CD8^+^ T cells residing in tumor tissues (44). Altogether, these studies suggest that IMs influence the behavior of immune effector cells and thus impact tumor progression and prognosis.

#### 4.2.3 A proinflammatory microenvironment

Oncobacteria, such as *Fusobacterium nucleatum* (Fn)*, Streptococcus gallolyticus*, and *Prevotella anaerobius*, induce the tumor inflammatory microenvironment by regulating inflammatory factor release and forming local inflammatory environments, such as NETs (41), thereby stimulating cancer cells to proliferate (45). In a liver metastasis murine model of CRC, orally administered *F. nucleatum* exerted deleterious effects on the distant tumor, along with substantially increasing plasma levels of proinflammatory cytokines, such as IL6, IL12, IL9, IL17A, CXCL1, monocyte chemoattractant protein-1(MCP-1), TNF-α, and IFN-γ as well as monocyte myeloid-derived suppressor cell (MDSC) accumulation, abridgment in NK and Th17, and a decrease in the α diversity of the gut microbiome, but elevating *Enterococcus* and *Escherichia/Shigella* levels (46). *H. pylori* induced*-*NF-κB activates the PIEZO1-YAP1-CTGF axis to reshape the gastric cancer microenvironment by promoting CAF infiltration, therefore targeting PIEZO1-YAP1-CTGF might be a potential therapeutic option to prevent gastric cancer (GC) progression and peritoneal metastasis (47).

As for fungi, *Aspergillus sydowii* (*A. sydowii*), enriched in lung adenocarcinoma (LUAD) tissue, provokes IL-1β secretion and its associated MDSC accumulation and expansion through the β-glucan/Dectin-1/CARD9 pathway, which suppresses cytotoxic T cells and ICI therapy (11). Likewise, microbiota residing in the lung mucosa spur myeloid cells release Myd88-dependent IL-1β and IL-23, resulting in Vγ6+Vδ1+γδ T cell proliferation and secretion of IL-17 and other cytokines to form an inflammatory microenvironment, thus accelerating the development of lung cancer due to KRAS mutation and p53 loss. This evidence seems to imply that ablating microbiota through antibiotic treatment is a good candidate. Interestingly, another study shared a different perspective. Mice treated with antibiotics are more susceptible to Lewis lung carcinoma because of defects in inducing the γδT17 cell response and subsequent IL-6 and IL-23 release. This may be a result of the differences in the pathogenesis of different lung cancers (48). In another example, fungi in PDAC elevate IL-33 secretion by tumor cells, further recruiting Th2 and innate lymphoid cells 2 (ILC-2), which then promotes tumor progression by releasing pro-tumorigenic cytokines such as IL-4/5/13 (56).

#### 4.2.4 A tumor immunosuppressive microenvironment

IMs and their metabolites can fail ICI or other therapeutics through immune escape and immune effector cell anergy. Previous studies have reported that IMs, such as HBV, HCV, *S. aureus*, and *N. ramosa*, enhance Treg immunosuppression in the TME, thereby mediating prostatic cancer (PCA) and HCC growth. As a result of inflammation and antiviral responses (49), advanced forms of HPV (+) tumors have a high infiltration of immune cells in the TME, however, the main content is myeloid cells instead of DCs and cytotoxic T cells, weakening anti-tumor activity (50). Microbiota within pancreatic cancer causes T cell disability through selective Toll-like receptor ligation, thereby leading to increased levels of MDSC and M2 macrophages to form a suppressive TME (57). Commensal fungi in breast cancer and melanoma can reduce T cells and increase M2-like TAMs by conjugation with Dectin-1, thus offsetting anti-tumor immunity (58).

#### 4.2.5 Complement and molecular mimicry

Glycans on the wall of intratumoral *Malassezia* spp. ligating with mannose-binding lectin (MBL) elicits complement cascades that promote tumor progression (51). Molecular mimicry is another mechanism for fungi to promote cancer. For instance, Candida albicans express complement receptor 3-related protein (CR3-RP), structurally similar to CR3 on the leukocytes, thereby disturbing the immune response in the TME (59).

Taken together, intratumoral microbes modulate anti-tumor immunity in multiple ways. Their functions depend on various factors, far beyond the IM composition, tumor type and status, and external factors including antibiotics and diet, which is worth exploring in clinical practice.

## 5 The signaling pathway of intratumoral microbiota in tumorigenesis and immunity

**Figure 3:**
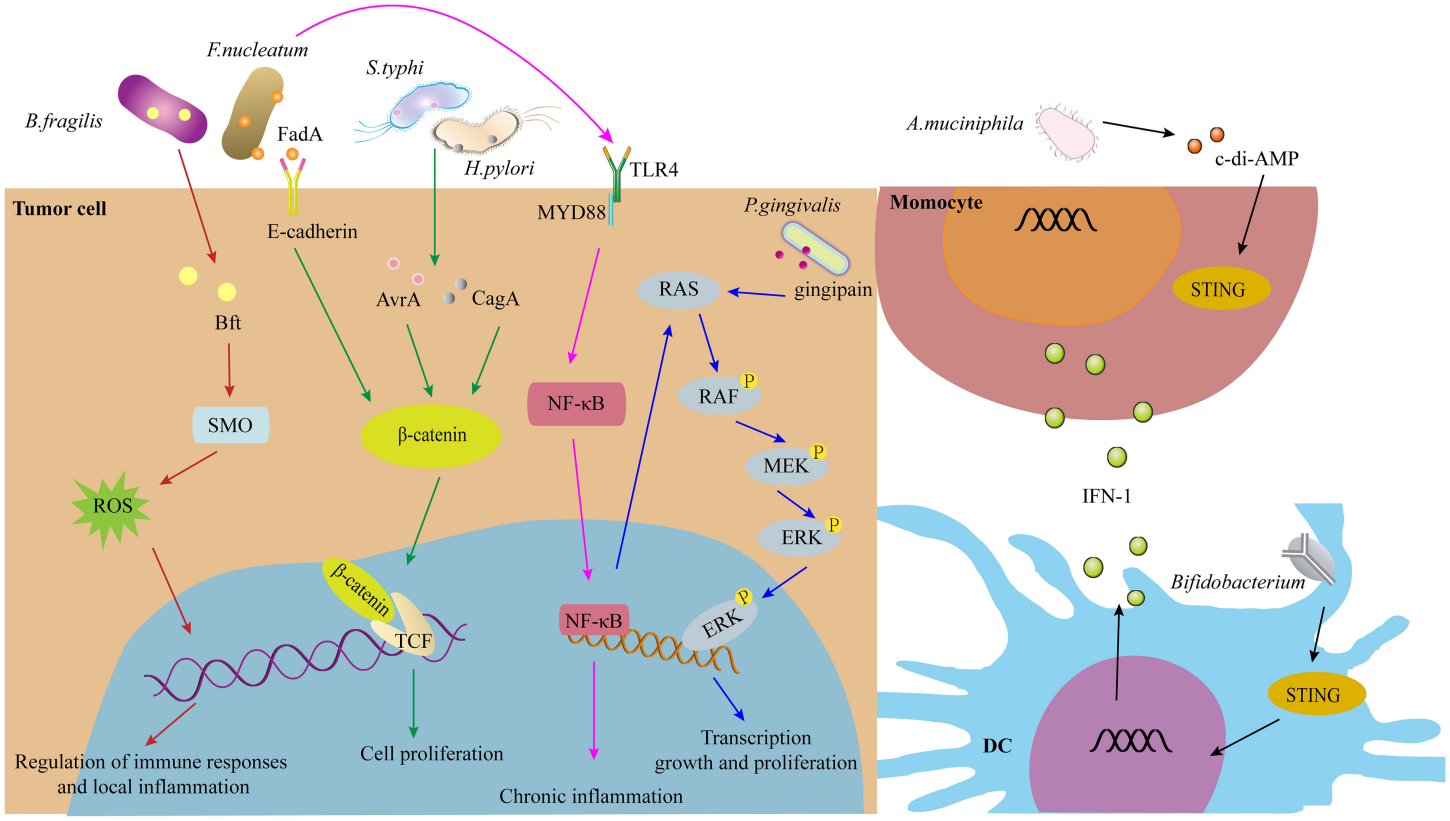
The signaling pathways of IMs in tumorigenesis and immunity, such as ROS, β-catenin, STING signaling pathway, and others.

As discussed previously, intratumoral microorganisms play a significant role in tumorigenesis and the regulation of anti-tumor immunity. The signaling pathways through which these microorganisms influence tumor progression and immune responses include but are not limited to, ROS, β-catenin, STING, and others (**Figure 3**).

### 5.1 ROS signaling pathway

ROS are highly bioactive molecules that, at low to moderate levels, serve as signal transducers that activate cell proliferation, migration, invasion, and angiogenesis. However, high levels of ROS can lead to cellular damage, including protein, nucleic acid, lipid, membrane, and organelle injuries, culminating in cell death, EMT, and metastasis (60). For instance, *Enterotoxigenic Bacteroides fragilis* (ETBF) induces DNA damage via ROS (3). In the gastrointestinal tract, *F. nucleatum* activates Toll-like receptor 4 (TLR4)-ROS signaling, promoting NET formation and thereby enhancing tumor progression and metastasis through angiogenesis, EMT, and matrix metalloproteinase (MMP)-mediated degradation of basement membrane proteins (41).

### 5.2 β-catenin signaling pathway

The Wnt/β-catenin signaling pathway plays crucial roles in embryonic development and adult tissue homeostasis. Dysregulation of this pathway contributes to various diseases, including cancer. *H. pylori* infection significantly increases cyclin-dependent kinase 1 (CDK1), which phosphorylates and inhibits GSK-3β activity, resulting in the accumulation and activation of β-catenin and NF-κB in gastric tumors (61). Additionally, β-catenin and yes-associated protein (YAP) synergistically promote *H. pylori-induced* gastric carcinogenesis by regulating downstream genes such as CDX2, LGR5, and RUVBL1 (62). Furthermore, FadA adhesin from *F. nucleatum* enhances Annexin A1 expression in Wnt/β-catenin signaling, forming a positive feedback loop through E-cadherin and activating Cyclin D1 specifically in cancerous cells, not in non-cancerous cells (63). *F. nucleatum* interaction with CDH1 triggers phosphorylation events that upregulate downstream β-catenin, Cyclin D1, and Myc to promote squamous cell carcinoma proliferation (64). In another study, the *F. nucleatum* group showed significantly increased expression of TLR4, PAK1, p-PAK1, p-β-catenin S675, and cyclin D1 compared to the control group, suggesting TLR4 as a potential therapeutic target for *F. nucleatum*-related colorectal cancer prevention and therapy.

### 5.3 STING signaling pathway

Cyclic GMP-AMP synthase (cGAS) functions by detecting misplaced genomic, mitochondrial, and microbial double-stranded DNA (dsDNA), leading to the synthesis of 2’3’-cGAMP. This molecule activates STING, thereby initiating innate immune responses. This mechanism serves as a pervasive and effective surveillance system against tissue damage and pathogen invasion. However, dysregulated cGAS-STING signaling plays a significant role in infectious, autoimmune, malignant, fibrotic, and neurodegenerative diseases (65). The cGAS/STING pathway can promote macrophage polarization, contributing to potent anti-tumor immunity (65). In tumor models, both systemic delivery and local administration of *Bifidobacterium* result in its accumulation within tumors, converting non-responders into responders in a STING- and interferon-dependent manner. Furthermore, *Bifidobacterium* enhances dendritic cell cross-priming following anti-CD47 treatment (66). In cancer immunotherapy, *Bifidobacterium* improves the efficacy of immune checkpoint inhibitors through STING and adenosine 2A receptor (A2AR) signaling pathways (67).

### 5.4 Other signaling pathways (NF-κB, TLR, complement, RhoA/ROCK, PERK)

In addition to the aforementioned pathways, intratumoral microbes may activate other signaling pathways such as NF-κB, TLR, complement, RhoA/ROCK, and PERK. For instance, *E. coli* colonization in colorectal cancer liver metastasis (CRLM) enhances lactate production, promoting M2-like macrophage polarization via NF-κB signaling facilitated by retinoic acid-inducible gene 1 (RIG-I) lactylation, thereby contributing to colorectal cancer liver metastasis (68). In both immunocompetent and immunodeficient melanoma mouse models, flagellum-deficient *Salmonella* fails to induce significant anti-tumor effects, despite higher bacterial inoculation, due to the absence of the Flagellin/Toll-like receptor 5 (TLR5) signaling pathway. Mannose-binding lectin (MBL) binds fungal glycans and activates the complement cascade, potentially mediating tumor progression. Intratumoral microorganisms also impact tumorigenesis and anti-tumor immune responses through other signaling pathways such as RhoA/ROCK and PERK (69–71).

## 6 Clinical implications of intratumoral microbiota

The intratumoral microbiota not only plays a role in tumor initiation, progression, and metastasis but also possesses significant clinical implications. These include targeting intratumoral bacteria for cancer therapy and utilizing intratumoral microbiota as diagnostic and prognostic tools.

### 6.1 Targeting the microbiota for cancer therapy

Microbe-based cancer therapies have shown efficacy, yet some IMs can induce an immunosuppressive microenvironment, leading to therapeutic failure and adverse effects. Therefore, strategies aimed at eliminating specific tumor-associated IMs are highly desirable. For instance, the administration of bismuth colloidal pectin granules to gastric cancer patients infected with *H. pylori* reduced side effects and improved symptoms (ClinicalTrials.gov Identifier: NCT05049902) (72). Additionally, the efficacy of itraconazole in treating various cancers has been explored in preclinical and clinical trials (ClinicalTrials.gov Identifier: NCT02749513) (73). Nevertheless, the use of antibiotics may result in flora imbalance and drug resistance. Future advancements may include the use of phage-based or engineered phage-based therapies targeting oncobacteria for effective tumor treatment.

### 6.2 Diagnostic and prognostic roles of intratumoral microbiota

The abundance of intratumoral microbes in different tumors, along with the presence of tumor type- and subtype-specific microbial profiles, suggests that IMs can serve as valuable biomarkers in cancer diagnostics and prognostics. For example, the intratumoral microbiome characteristics of HNSCC are associated with clinicopathological features such as tumor stage and histological grade. A recent study revealed that the microbiome in GC can be classified into three microbial subtypes (MS1, MS2, and MS3), each with distinct characteristics that are linked to immunotherapy and prognosis (74). Another study found that the relative abundance of viruses in soft tissue sarcomas (STS) is positively correlated with higher NK cell infiltration, which is associated with improved metastasis-free and overall survival (75). In pancreatic cancer, the presence of *F. nucleatum* and *P. gingivalis* is associated with a higher risk and worse prognosis (76). High levels of *F. nucleatum* are particularly correlated with the stage and recurrence of esophageal squamous cell carcinoma (ESCC) (77). These findings underscore the potential of intratumoral microbiota as biomarkers in cancer diagnostics and prognostics.

## 7 Applications in therapeutics

**Figure 4:**
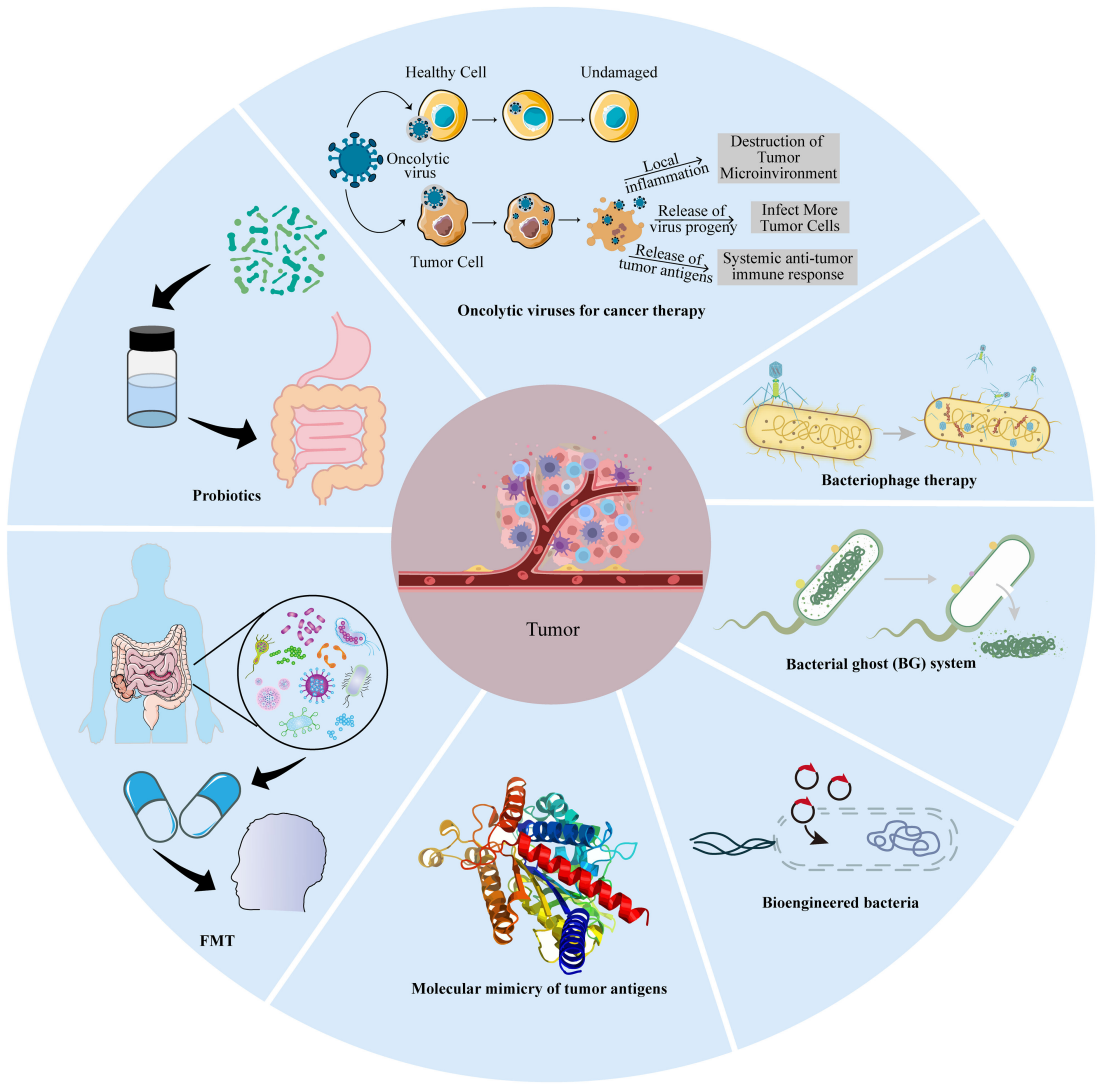
Microbial-based cancer treatment strategies. These include oncolytic virotherapy (OVT), bacteriophage therapy, bacterial ghost (BG), bioengineered bacteria, molecular mimicry, fecal microbiota transplantation (FMT), and probiotics.

Based on the roles of intratumoral microbiota in tumors, the ablation of pro-tumoral IMs and supplementation with tumor-hostile IMs are feasible approaches therapeutically (78), but fecal microbiome transplantation (FMT), probiotic regulation, microbial peptide-specific T cell universal cancer vaccines, and engineered microbes may have more prospects for cancer therapy (**Figure 4**).

### 7.1 Fecal microbiome transplantation

FMT can effectively treat recurrent antibiotic-resistant infections caused by *C. difficile*, which has been evaluated in inflammatory bowel disease (IBD) (79). Baruch and coworkers reported that 10 patients with metastatic melanoma who were resistant to ICIs were treated with vancomycin and neomycin for 3 days to ablate native microbiota before FMT via both colonoscopy and administration of oral stool capsules. FMT products were extracted from two donors with a complete response to nivolumab for metastatic melanoma for over 1 year. Three patients (3/10)overcame ICI resistance after FMT, one with a complete response, and two with partial responses (NCT03353402) (80). In two independent clinical trials (NCT03341143 and NCT03772899), FMT from long-term responder donors with ICI-refractory advanced melanoma alone showed effective clinical responses or prolonged survival without serious side effects. FMT ameliorates the dysbiosis of bacterial flora associated with anti-PD-1 clinical responses and increases anti-tumor immunity (81, 82). Although favorable and unfavorable species of bacteria have been identified, the ingredients of an “ideal gut microbiota” remain elusive. Thus, there is not a one-size-fits-all gut microbiota. FMT faces several main challenges: ① The suitable microbiota composition for donors is not yet established at present. ② There are no available screening methods or prognostic factors that can be harnessed to select candidate recipient patients for FMT plus ICI treatment. ③ FMT failure is likely attributed to patients’ non-response to immunotherapy, tumorigenesis unrelated to the gut microbiome, a non-favorable microbiome in FMT for boosting targeted therapy, or recipient non-compliance with the donor’s microbiome. ④ The incidence of antibiotic resistance and pathogenic transmission in practical applications are further challenges. For this reason, FMT is not an approved treatment by either the Food and Drug Administration (FDA) or European Medicines Agency (EMA). Together, these early findings have important implications for modulating the gut microbiota in cancer treatment. In the future, the modulation of the gut microbiota, particularly with FMT regimens (delivery routes, frequency, duration, antibiotic pre-treatment) should select donors and recipients to improve the likelihood of success.

### 7.2 Probiotic regulation

Probiotics can survive or temporarily colonize the intestine, which is an important mechanism to manipulate the gut microbiota to increase the number of “beneficial bacteria.” Probiotic-based manipulation of the gut microbiota inevitably affects host metabolism, immune function, and digestion (83). At present, some species of bacteria have been utilized as probiotics, such as *lactic acid bacteria*, *Bifidobacterium*, and *Clostridiales* (83), to treat various diseases, including diarrhea, IBD, rheumatoid arthritis, chronic sinusitis, and cancer (84, 85). Mice administered with cisplatin in combination with *Lactobacillus* exhibited superior outcomes, including reduced tumor size and increased survival rates, compared to mice receiving cisplatin alone or cisplatin plus antibiotics. This suggests that *Lactobacillus* may mitigate the adverse effects commonly associated with chemotherapy (86). Radiation-induced intestinal injury is a common complication following abdominal or pelvic radiotherapy. The intervention with *Lactobacillus rhamnosus GG* (LGG) improves intestinal structure, reduces jejunal DNA damage, and inhibits the inflammatory cGAS/STING pathway, suggesting a potential therapeutic radioprotective effect (87). *Lactobacillus acidophilus* combined with *Bifidobacterium bifidum* could reduce the incidence of radiation-induced diarrhea and the necessity for anti-diarrheal medication in cervical cancer patients undergoing radiotherapy, with significant improvements in stool consistency (trial registration number: TCTR20170314001) (88). Members of the *Clostridiales* order within the gut microbiome, which have been linked to a reduced tumor burden, were found in a mouse model of CRC. Furthermore, these beneficial species were observed to be sharply diminished in CRC patients compared to their healthy counterparts. Notably, treatment with four strains of *Clostridiales* (CC4) or individual strains demonstrated an ability to impede or effectively treat CRC as a monotherapy. In a head-to-head comparison with anti-PD-1 therapy, supplementation with a mixture containing CC4 strains exhibited superior efficacy in mouse models of CRC and MM (24). Accumulation of *Bifidobacterium* within tumors reversed anti-CD47 treated mice from non-responsive to responsive via potently stimulating STING and IFN-I signaling as aforementioned (66). These studies provide not only pre-clinical evidence for harnessing FMT and probiotics as a monotherapy for solid tumors but also some limitations in practical applications (89). One study revealed that the dietary tryptophan catabolite indole-3-aldehyde (I3A), produced by the colonizing probiotic *Lactobacillus reuteri* (Lr), locally promotes IFN-γ-producing CD8^+^ T cells, thereby sustaining the efficacy of ICIs. The absence of Lr-secreted I3A or impaired aryl hydrocarbon receptor (AhR) signaling within CD8^+^ T cells negates the anti-cancer effects of Lr. Chimeric antigen receptor (CAR)-T cells have demonstrated remarkable success in treating hematological malignancies but have encountered limited efficacy against solid tumors (90). A recent study unveiled that tumor-colonizing probiotics release synthetic targets that mark tumor tissue for CAR-T-mediated cytolysis *in situ*. The innovative probiotic-guided CAR-T cells (ProCARs) system can activate and direct CAR-T cell activity for antigen-agnostic cell lysis across various cancer models (91). These breakthroughs highlight the potential of intratumoral microbiota manipulation as a complementary strategy to conventional and immunotherapeutic approaches, offering a beacon of hope for improved therapeutic outcomes in cancer treatment.

### 7.3 Microbial peptide-specific T cell universal cancer vaccines

Molecular mimicry (92–94) of tumor antigens homologous to microbial epitopes supplies cross-reactive T cells with ideal targets (95). Besides this paradigm, tumor cells are also capable of presenting microbial peptides directly to induce microbial-specific T cells, such as in MM (96). These peptides partially overlap with the HLA of tumor cells infected with coequal microbial strains.

These studies could usher in the development of microbial peptide-specific T cell universal cancer vaccines. Although the limited presence (0.1-10%) of bacteria in tumor cells (8, 69) may restrict its therapeutic efficacy, targeting bacterial peptides elicits greater anti-tumor immunity (by antigen dissemination) and facilitates a more precise tumor-specific T cell migration to the tumor site that is difficult for other methods to achieve. For instance, *F. nucleatum*-specific T cells could elicit anti-tumor immunity at the site of primary and metastatic tumors (43). In another example, one bacterial peptide called azurin-28 showed acceptable toxicity and tumor-inhibiting effects in pediatric brain tumor patients (97, 98).

The concept mentioned above is not novel, as previous attempts have been made to inject attenuated *Salmonella* directly into tumors over the past two decades in multiple cancer types (99–101). Recently, *Salmonella* infection in MM induced the unfolded protein response (UPR), leading to the release of peptides from tumor cells to generate anti-tumor responses *in vivo* (102). This new antigenic peptide can be further applied as a universal vaccine platform. Although *Salmonella* is not regarded as an intratumoral microorganism, this work provides new approaches for cancer therapy.

### 7.4 Bioengineered bacteria

Although bacteria can display immunostimulatory features and possess the potential for cancer therapy, bioengineered bacteria can acquire more versatile functions to achieve better efficacy through synthetic biology, which selectively and preferentially colonize in hypoxic and necrotic TMEs. These bioengineered bacteria, including *S. typhimurium*, *Listeria Monocytogenes, Bifidobacterium longum, Lactobacillus species*, and *Clostridium* species, have been utilized as delivery vectors with no serious adverse effects. In *S.typhimurium*, two chromosomal genes were deleted, namely, *pur*I and *msb*B, to reduce toxicity and increase safety (103). Recently, attenuated *Salmonella typhimurium* VNP20009 was engineered to exploit its tumor-targeting capabilities and induce the expression of GM-CSF and IL-7 in response to L-arabinose (named as GM-CSF-IL-7-VNP20009). Intravenous inoculation of GM-CSF-IL-7-VNP20009 in mouse models of subcutaneous and lung metastatic melanoma resulted in significant inhibition of melanoma growth and metastasis and prolonged the survival of the mice by significantly increasing the number of tumor-infiltrating macrophages and mature DCs, and elevated the proportion of CD4^+^ T cells as well as their proliferation in cancers (104). Currently, an *L.monocytogenes*-based cancer vaccine named ADXS11-001 is in several Phase II clinical trials to evaluate its efficacy for HPV-associated cancers (NCT02853604) (105). Additionally, different probiotic bacteria including *Bifidobacterium longum* and *Lactobacillus plantarum* are regarded as anticancer-targeting vectors. *Clostridia* strains such as *C. acetobutylicum, C. beijerinckii*, and *C. sporogenes* could effectively be modified to express some bacterial enzymes (nitroreductase, cytosine deaminase) or murine-TNFα to enhance the efficacy of cancer treatment (106). *Clostridium perfringens* exhibits antitumor activity due to its enterotoxin to damage the tight junction in the epithelial cells (107). In a clinical trial (NCT01924689), a single intratumoral injection of *C. novyi-NT*, which lacked the alpha toxin, for patients with injectable, treatment-refractory solid tumors can generate bacterial spore germination in the tumor, and leads to the killing of tumor cells and an inflammatory response with significant but controllable toxicities (108). An engineered *E. coli* Nissle 1917 strain can colonize tumors and convert ammonia to increase intratumoral L-arginine levels which increases the number of tumor-infiltrating T cells and has significant synergistic effects with PD-L1 blocking antibodies to clear tumors (109). Bacteria engineered to synthesize CD47 nanobodies could avoid adverse side effects caused by an unwanted CD47 blockade of red blood cells and platelets (110). Intratumoral injection of engineered *Lactococcus lactis* strains expressing Fms-like tyrosine kinase 3 (Flt3) ligand and OX40 ligand into one flank in mice bearing tumors bilaterally caused regression of tumors on both sides and increased anti-PD1 efficacy by 40% when the two methods were combined, although on the opposite side, after draining lymph nodes and the whole body, viable bacteria were not detected by the culture method (111). The results of these engineered bacteria to improve the response rates are exciting. However, one question remains: can certain bacteria survive for a period of time in a certain TME? Strange infiltration and proliferation behavior of IMs can occur in xenograft tumor models. *Salmonella typhimurium* is capable of colonizing BXPC-3 cells and slowly proliferating in the HCT116 model, whereas *E. coli* cannot in BXPC-3 cell-bearing tumors (112). In 1990, the FDA approved BCG for perfusion treatment of superficial bladder cancer, and BCG perfusion treatment remains the standard first-line treatment for bladder cancer (113).

Additionally, the bacterial ghost (BG) system is another innovative bacteria-based cancer therapy, which lacks ribosomes, nucleic acids, and some more intracellular constituents. The drugs, DNA, proteins, enzymes, and other therapeutic agents can fill the inner space of the BGs (114). For instance, cancer cell lysate (oncolysate)-loaded BGs induced by IFN-α and GM-CSF enhances DC maturation to increase the expression of maturation markers and co-stimulatory molecules, produce higher IL-12 levels, and significantly increase the proliferation of CD4^+^ and CD8^+^T cells compared to DCs in the presence of LPS (115). Bacteria-derived vesicles are also utilized in cancer therapy. Liu and coworkers constructed a bacteria-derived outer membrane vesicle (OMV)-coated nanoplatform that reached the dual-target kill of pre-tumor *F. nucleatum* and tumor cells, thus converting intratumoral bacteria into immunopotentiators in immunotherapy of triple-negative breast cancer (TNBC) (116).

Apart from the above advantages, engineered bacteria also face some obstacles in clinical applications, including bacterial virulence, anti-tumor capacity, precise targeting and colonization of tumors, genetic instability, impacts on the environment, and drug tolerance.

### 7.5 Bacteriophage therapy

Tumors are non-responsive to immunotherapy (117, 118), which may be due to the effects of human microbiota on inflammation and immune dysregulation (119). Although antibiotic therapy (ABT) can control intratumoral bacteria, it has detrimental effects on the microbiome and leads to antibiotic resistance (26). Moreover, some types of antibiotics increase the incidence of CRC, and ABT and tumor location are significantly correlated (120). Additionally, the broad-spectrum activity of antibiotics may not be able to distinguish whether bacteria are cancer-promoting or cancer-suppressive within the TME.

Bacteriophages are an attractive biomedical tool for therapeutic applications owing to their nanosize, polyvalent surface properties, non-pathogenic nature, and specifically infecting particular bacteria species and are receptive to desirable chemical or genetic modifications. Bacteriophages are able to serve as both a cargo-loaded device and self-immune adjuvant (45, 121), which makes targeting oncobacteria possible. For example, a specific phage may eliminate *S. gallolyticus* from cancer patients while retaining other anti-tumorigenic *Streptococci* (122). More *H. pylori* strictly virulent phages need to be isolated for therapy of chronic gastritis, peptic ulcer disease, and gastric cancer (123).

In the TME, phages can eliminate specific oncobacteria, leading to other tumorigenic bacteria growing (124). Furthermore, due to their adhesive and invasive toxicity, pro-tumoral bacteria such as *Fusobacterium* are present in biofilms or cells and evade the immune system (125). When utilizing phages as a therapy to restore microbiota from deviation, it is crucial to ascertain how they interact with each other within the niche.

Similar to engineered bacteria, bacteriophages can be genetically manipulated to improve cancer therapy (126, 127). For instance, the filamentous bacteriophage fUSE5-ZZ has been modified genetically and chemically to deliver anti-ErbB2 and anti-ERGR antibodies or the anti-tumor agents hygromycin and doxorubicin, for anti-tumor treatment (128). Likewise, engineered phages targeting *F. nucleatum* enhanced the effects of chemotherapy and improved survival in mouse and piglet CRC models by modifying them to covalently bind and optimize irinotecan delivery (124, 129).

Multiple phage mixtures have more potential than individual phages and can be prepared from a phage library, formulated, and administrated to some patients to modulate IMs and enhance cancer therapy (130–132). Recently, eight phages were engineered with tail fibers and clustered regularly interspaced short palindromic repeats (CRISPR)-Cas machinery to specifically target *E. coli* in biofilms. Furthermore, SNIPR001, composed of the four most complementary bacteriophages, is well tolerated and reduces *E. coli* load in the mouse gut better than its individual components. SNIPR001 can selectively kill *E. coli* which may lead to fatal infections associated with hematological cancer (133). Of note, before using phage therapy, it is necessary to accurately distinguish pro-tumoral stains from anti-neoplastic ones.

Though bacteriophages for cancer therapy mentioned above have been discussed, there are a few obstacles yet to be addressed during circulation and after reaching the tumor. Once injected into the bloodstream, bacteriophages could be cleared from circulation by the reticuloendothelial system (RES), which impacts their half-life, bio-distribution, and pharmacokinetics, which compromises the efficacy and may result in non-specific side effects. More importantly, biochemical and biophysical barriers prevent phages from entering into tumor sites. The major obstacle encountered by phages upon reaching the tumor site is related to diffusion. In the future, it is necessary to screen phages to target tumorigenic bacteria. Phages are modified to avoid clearance by the RES, prolong their half-life, and target tumors. It is necessary to better understand how to reduce the immune response-mediated phage clearance from the system. In addition, it is also important to optimize the use of bacteriophages for cancer therapy.

### 7.6 Oncolytic viruses for cancer therapy

Oncolytic viruses (OVs) are natural or genetically modified viruses that can specifically attack and destroy tumor cells. Generally, compared with “cold” tumors, “hot” tumors feature a high number of mutations, increased expression of tumor-infiltrating lymphocytes (TILs) and PD-L1, and response well to PD-1/PD-L1 inhibitors. Currently, there are no accurate methods to screen “cold” and “hot” tumors. Fortunately, oncolytic viruses, as a way to tweak the TME, can enhance the immune response, in combination with other treatments such as immune checkpoint blockade (ICB) (134–138). The most oncolytic viruses are those armed with targeting or functional factors, depending on the inserted gene sequences (139–143). Talimogene laherparepvec (T-Vec), a genetically modified attenuated HSV1 containing granulocyte-macrophage colony-stimulating factor (GM-CSF), is the first FDA-approved OV product to be administrated via intratumoral injection for postoperative recurrent unresectable MM. Several Phase II-III clinical trials of T-Vec for melanoma and other cancers are currently underway(NCT02779855, NCT00769704) (144, 145). There are also some oncolytic viruses being trialed alone or in combination with other treatments(NCT03152318; UMIN-CTR Clinical Trial Registry UMIN000015995; NCT03178032;NCT03072134) (136, 146–148). Various attempts have been made to modify OVs, including multi-gene, microRNA, and gene circuit insertion. Intratumorally inoculating with OVs arms a PD-L1 inhibitor and GM-CSF and fights against both virus-injected and distant tumors by reversing PD-L1-mediated resistance, priming T cell immune responses against mutation-derived dominant and subdominant neoantigen epitopes (149). The engineered measles virus expressing the synthesized microRNA universal cassette expressed the virally encoded microRNA within the endogenous microRNA transcripts and successfully inhibited the target protein (150). The replication and release of an adenovirus selectively in HCC cells can be regulated via programmable and modular synthetic gene circuits responding to multiple promoter and microRNA inputs, and it has superior cytolytic efficacy compared to non-replicable ones (151). Unlocking the potential of oncolytic virotherapy (OVT) appears to have bright prospects for cancer immunotherapy.

**Table 2** gives a comparison of the advantages and limitations of the therapeutic methods mentioned above. Dozens of IM products are being tested in clinical trials (**Table 3**), some of which have been approved by the FDA for clinical cancer therapy (**Table 4**). Additionally, microbe-based therapy combined with conventional therapies, and immunotherapy such as ICI or CAR-T, can increase efficacy or mitigate side effects. Clinical trials of microbe-based or combined therapy are listed in **Table 4**.

**Table 2:** Comparing the advantages and limitations of therapeutic methods mentioned in the text.

Methods	Advantages	Limitations
FMT/ Probiotics	① Extensive resources ② More acceptable ③ Suitable for the treatment of diverse other diseases ④ Decrease side effects ⑤ Turn non-responders to responders to immunotherapy	① Patients’ non-response to immunotherapy ② Tumorigenesis unrelated to the gut microbiome ③ Non-favorable microbiome in FMT for boosting targeted therapy ④ Recipient non-compliance with the donor’s microbiome ⑤ Imprecision ⑥ Antibiotic-resistance ⑦ Pathogenic transmission
Microbial peptide-specific T-cell universal cancer vaccines	Tumor-specific	① Expensive ② Peptides difficult to be found
Bioengineered bacteria, BG, and bacteria-derived vesicles	① More versatile ② Conveniently modified (insertion of cytokines/chemokines/antigens/antibodies or gene circuits) ③ Tumor-specific ④ Load with various biomacromolecules and drugs	① Survival period ② Bacterial virulence versus anti-tumor capacity ③ Precise targeting and colonization of tumors ④ Genetic instability ⑤ Impact on the environment, drug tolerance, and etc. ⑥ Expensive
Bacteriophage therapy	① Specifically kill oncobacteria ② A cargo-loaded device ③ A self-immune adjuvant ④ Mixture application ⑤ Conveniently modified	① Other tumorigenic bacteria growing instead ② Original tumorigenic bacteria not eliminated ③ Difficulty to distinguish tumor-friendly bacteria from tumor-harmful bacteria beforehand ④ relatively short half-life ⑤ non-specific side effects ⑥ diffusion
Oncolytic viro-therapy	① Specifically attack and destroy tumor cells ② Tweak the TME and activate anti-tumor immunity ③ Enhancing the efficacy of other anti-tumor agents in combination with OVs ④ Conveniently modified	① *In vivo* pre-existing neutralizing antibody ② Lower efficacy of OVs alone ③ Poor targeting delivery efficacy

FMT, Fecal microbiome transplantation; IM, Intratumoral microbiota; OV, Oncolytic virus.

**Table 3:** Clinical trials of anti-tumor microbe-based therapies.

Microbe-based therapy	Tumor type	Clinical Studies	Database (ID)/FDA approval	Reference
FMT	Leukemia	Prevention of dysbiosis complications with autologous FMT in AML Patients	NCT02928523	(152)
Intestinal microbiome	Gastric Cancer	The recovery of Intestinal Microbiome after gastrectomy	NCT03418428	(153)
Probiotics (Bacteria)	HCC	Influence of probiotics administration before liver resection in liver disease	NCT02021253	(154)
Bl-04, NCFM (Bacteria)	Colon cancer	Using probiotics to reactivate tumor suppressor genes in colon cancer	NCT03072641	(155)
Bifidobacterium longum	HCC	The significance of LFR on patient long-term survival through retrospective and prospective cohorts and identified a key gut microbe, Bifidobacterium longum, depleted in patients with delayed recovery.	NCT05178524	(156)
C. novyi-NT	Injectable, treatment-refractory solid tumors	This first-in-human study enrolled patients with injectable, treatment-refractory solid tumors to receive a single intratumoral injection of C. novyi-NT across 6 dose cohorts to determine dose-limiting toxicities (DLT), and the maximum tolerated dose.	NCT01924689	(108)
ADXS11-001(*L. monocytogenes*)	Cervical cancer	Phase 2 study evaluated the safety and efficacy of ADXS11-001, administered with or without cisplatin, in patients with recurrent/refractory cervical cancer following prior chemotherapy and/or radiotherapy.	Clinical Trials RegistryYIndia (CTRI/2010/091/001232)	(157)
JX-594 (Oncolytic virus)	HCC	To determine the optimal JX-594 dose in subjects with advanced hepatocellular carcinoma (HCC)	NCT00554372	(158)
	metastatic melanoma	A mechanistic proof-of-concept clinical trial was performed at a low dose equivalent to ≤10% of the maximum-tolerated dose (MTD) in other clinical trials	NCT00429312	(159)
	refractory colorectal cancer	The primary endpoint was to determine the maximum tolerated dose. Secondary endpoints were pharmacokinetics and pharmacodynamics as well as antitumor activity.	NCT01469611	(160)
	liver cancer	Assess intratumoral injection of JX-594 in patients with refractory primary or metastatic liver cancer	NCT00629759	(161)
T-VEC	Melanoma	Stage IIIc and Stage IV Malignant Melanoma	NCT00769704	(145)
G207 (Oncolytic virus)	Brain Cancer	a phase 1 trial of G207, which used a 3 + 3 design with four dose cohorts of children and adolescents with biopsy-confirmed recurrent or progressive supratentorial brain tumors.	NCT02457845	(162)
G47∆ (a triple-mutated, third-generation oncolytic herpes simplex virus type 1)	glioblastoma	This investigator-initiated, phase 2, single-arm trial primarily assessed the efficacy of G47∆, a triple-mutated, third-generation oncolytic herpes simplex virus type 1, in 19 adult patients with residual or recurrent, supratentorial glioblastoma after radiation therapy and temozolomide.	Clinical Trial Registry UMIN000015995	(147)
G47∆	glioblastoma	a phase I/II, single-arm study assessing the safety (primary endpoint) of G47∆, a triple-mutated oncolytic herpes simplex virus type 1, in Japanese adults with recurrent/progressive glioblastoma despite radiation and temozolomide therapies.	UMIN-CTR Clinical Trial Registry UMIN000002661	(163)
Enadenotucirev (Oncolytic virus)	CRC, NSCLC, UCC RCC;	Assessed intravenous (IV) delivery of enadenotucirev in patients with resectable colorectal cancer (CRC), non-small-cell lung cancer (NSCLC), urothelial cell cancer (UCC), and renal cell cancer (RCC) with a comparator intratumoral (IT) dosed CRC patient cohort;	NCT02053220	(164)
	advanced epithelial tumors unresponsive to conventional therapy	This phase 1 dose escalation study assessed intravenous (IV) dose escalation with enadenotucirev to establish the maximum tolerated dose (MTD) and subsequently identify a suitable schedule for repeated cycles.	NCT02028442	(165)
V937, a novel OV non-genetically modified Kuykendall strain of Coxsackievirus	unresectable melanoma	Evaluated the activity of intratumoral Coxsackievirus A21 (V937) in 57 patients with unresectable stage IIIC or IV melanoma.	NCT01227551; NCT01636882	(166)
CAN-3110, an oncolytic herpes virus (oHSV)	recurrent glioblastoma (rGBM)	intralesional oHSV treatment enhances anticancer immune responses even in immunosuppressive tumour microenvironments, particularly in individuals with cognate serology to the injected virus.	NCT03152318	(146)
T-VEC +nivolumab	resectable early stage or metastatic (IIIB-IVM1a) melanoma	a single center, single arm, phase II study aims to show an improved major pathologic complete response (pCR) rate, either pCR or near-pCR, up to 45% in 24 patients with resectable stage IIIB-IVM1a melanoma upon neoadjuvant combination treatment with intralesional T-VEC and systemic nivolumab (anti-PD-1 antibody).	NCT04330430	(167)
T-VEC+neoadjuvant chemotherapy	TNBC	T-VEC can enhance triple-negative breast cancer (TNBC) responses to neoadjuvant chemotherapy (NAC).	NCT02779855	(144)
T-VEC+ pembrolizumab	melanoma	The efficacy and safety from a phase III, randomized, double-blind, multicenter, international study of T-VEC plus pembrolizumab (T-VEC-pembrolizumab) versus placebo plus pembrolizumab (placebo-pembrolizumab) in patients with advanced melanoma.	NCT02263508	(168)
DNX-2401 + pembrolizumab	recurrent glioblastoma	overall safety and objective response rate	NCT02798406	(169)
V937 + pembrolizumab intravenous	melanoma	CAPRA evaluated Coxsackievirus A21 (V937) + pembrolizumab for metastatic/unresectable stage IIIB-IV melanoma.	NCT02565992	(170)
V937 ± pembrolizumab	solid tumors	This phase 1 study evaluated intravenous V937 ± pembrolizumab in patients with advanced solid tumors.	NCT02043665	(171)
V937 + ipilimumab	melanoma	an open-label, single-arm, phase 1b study (NCT02307149) evaluating V937 plus the cytotoxic T-lymphocyte antigen 4 inhibitor ipilimumab in patients with advanced melanoma.	NCT02307149	(172)
V937+ ipilimumab	uveal melanoma	The phase 1b, open-label CLEVER study evaluated V937 in combination with ipilimumab in patients with uveal melanoma	NCT03408587	(173)
adenovirus enadenotucirev+nivolumab	epithelial cancer	a phase I multicenter study of intravenous enadenotucirev plus nivolumab in patients with advanced/metastatic epithelial cancer not responding to standard therapy.	NCT02636036	(174)

**Table 4:** FDA-approved drugs and products using IMs in anti-cancer and immune therapy.

IMs products	Category	Cancer type	Year approved by FDA
BCG	Bacillus Calmette-Guérin (BCG), attenuated vaccine made from *B. tuberculosis bovis*	Superficial bladder cancer via perfusion therapy	1990
T-VEC	an oncolytic herpes virus1 (oHSV1) carrying with GM-CSF	Local treatment of unresectable cutaneous, subcutaneous, and lymph node lesions in patients with recurrent melanoma after initial surgery	2015
CG0070	Adenovirus 5 vector embedded two coding genes-tumor-selective promoters E2F-1 and GM-CSF genes.	high-risk, non-muscle invasive bladder cancer (NMIBC) BCG therapy does not respond.	2023
BCG plus N-803	Bacteria and heterodimeric IL-15 agonists	BCG-unresponsive bladder CIS with or without Ta/T1 papillary disease.	2024
SGN1	Oncolytic bacteria, attenuated Salmonella vector carrying a specific methionine hydrolase	hepatocellular carcinoma	2024
CAN-3110	an oncolytic herpes virus (oHSV) deleted or removed the viral gene encoding ICP34.5	Recurrent high-grade glioma	2024

## 8 Conclusions and perspectives

In this review, we have outlined how the composition and diversity of intratumoral microbiota influence tumor initiation, development, and metastasis, as well as anti-tumor immunity through signaling pathways including ROS, Wnt/β-catenin, and STING, with significant clinical implications. We have also proposed strategies for microbe-based cancer therapy. Despite being recognized as a “hallmark” of cancer, research in this field remains nascent, with numerous unresolved issues. For example, the role of intratumoral phages remains largely unexplored. How do bacteria, fungi, viruses, and other microbes within the TME interact to influence tumor initiation, progression, and anti-tumor immune responses? Determining whether specific intratumoral microorganisms act as cancer promoters or tumor suppressors remains a challenge. Additionally, issues surrounding the precision and resistance of FMT or bacterial transplantation need resolution. The systemic administration of engineered bacteria or oncolytic viruses to target tumor tissues also presents considerable challenges.

Further research is needed to elucidate how intratumoral microbes can be leveraged to enhance the efficacy of immunotherapy and mitigate the cytokine storm and neurotoxicity induced by CAR-T cell therapy. With advancements in technologies such as multi-omics, synthetic biology, and artificial intelligence (AI), our understanding of the roles of intratumoral microbiota will improve, leading to the emergence of new concepts and innovative cancer strategies and therapeutic agents. Microbe-based therapies can be used independently or in combination with current immunotherapeutic approaches to enhance efficacy, predict treatment outcomes, or mitigate the toxicity associated with treatments such as CAR-T cell therapy and ICB, thereby advancing effective cancer therapy.
